# Prospective immune profiling in critically ill adults: before, during and after severe sepsis and septic shock

**DOI:** 10.1186/cc14123

**Published:** 2015-03-16

**Authors:** N Layios

**Affiliations:** 1 CHU Sart Tilman, Liège, Belgium

## Introduction

Rethinking the host's defense mechanisms during severe infection has led to the use of flow cytometry (FCM) and to the current concept of sepsis-induced immunosuppression. However, organ dysfunctions that develop in the period preceding severe sepsis as a consequence of surgery, trauma or burn might also trigger immune reprogramming predisposing to overwhelming infection. Our aim was to look for correlation of specific phenotypes among four commonly encountered populations of patients and the later occurrence of severe sepsis and septic shock.

## Methods

In total, 114 non-infected patients were prospectively screened via FCM on days 1 (T1) and 3 (T2) of elective cardiac surgery, trauma, acute neurologic dysfunction and prolonged ventilation (>48 hours). A third sample was drawn when infection was diagnosed (Tx) and 7 days later (Tx + 7). Exclusion criteria included use of immunosuppressive agent(s). The broad panel of cell-specific antibodies focused on B, T lymphocytes (Tregs, Th17, NKT), NK cells, monocytes and neutrophils. Plasmatic levels of IL-2/IL-6/IL-7/TNFα/ IFNγ were also determined.

## Results

Ninety-nine patients were included in the final analysis. Eighteen patients developed severe sepsis or septic shock. They presented with significantly higher levels of intermediate (CD14^++^/16^+^) and CD62L^- ^monocytes and lower IL-2 levels at T1 compared with patients who did not get septic. ROC AUC for association of these parameters with the occurrence of sepsis were 0.78 (95% CI: 0.63 to 0.91), 0.72 (0.62 to 0.82) and 0.73 (0.65 to 0.82), respectively. High counts of these monocytic cells were also associated with increased 90day mortality (*P *< 0.01, ROC AUC = 0.87 (0.77 to 0.95), 0.79 (0.66 to 0.9)). Kaplan-Meier survival curves showed significantly higher mortality after stratification based on these cell counts at T1 (CD14^++^CD16^+^: cutoff >236.8 cells/μl, HR = 23.6 (*P *= 1.24 × 10^-5^); CD62L^-^: cutoff >95.4 cells/μl, HR = 6.67 (*P *= 7.6 × 10^-4^)). Multivariate logistic regression analysis using clinical scores indicated that addition of IL-2 levels at T1 significantly improved prediction of sepsis (OR = 0.834, *P *= 0.02).

## Conclusion

Predisposition to sepsis in selected critically ill medico-surgical adults can be identified on day 1 of admission based on high counts of circulating intermediate and CD62L^- ^monocytes and low levels of IL-2 (the latter provide incremental prognostic information). High counts of these specific monocytes correlate with higher 90-day mortality.

