# Erratum: Prospective immune profiling in critically ill adults: before, during and after severe sepsis and septic shock

**DOI:** 10.1186/s13054-015-1006-6

**Published:** 2015-08-26

**Authors:** N. Layios, C. Gosset, C. Delierneux, A. Hego, J. Huart, A. Joly, P. Geurts, P. Damas, C. Oury, A. Gothot

**Affiliations:** Department of General Intensive Care, University Hospital Centre of Liège, Domaine Sart-Tilman B35, Liège, 4000 Belgium; GIGA-Cardiovascular Sciences, Laboratory of Thrombosis and Hemostasis, University of Liège, Domaine Sart-Tilman B35, 4000 Liège, Belgium; CHU de Liège, Domaine Sart-Tilman B35, 4000 Liège, Belgium; Laboratory Hematology, University Hospital Centre of Liège, Liège, Belgium; Systems and Modeling, Department of Electrical Engineering and Computer Science and GIGA-R, University of Liège, Domaine Sart-Tilman B35, 4000 Liège, Belgium

## Erratum

After publication of our recent Poster presentation [1], we noticed that C Gosset , C. Delierneux, A. Hego, J Huart, A Joly, P Geurts, P Damas, C Oury and A Gothot had been inadvertently omitted as co-authors. The author list is now complete and the competing interests section modified accordingly.
